# Use of the supportive care framework to explore haematological cancer survivors’ unmet needs: a qualitative study

**DOI:** 10.1186/s12913-020-05927-7

**Published:** 2020-11-23

**Authors:** Anne Herrmann, Elise Mansfield, Flora Tzelepis, Marita Lynagh, Alix Hall

**Affiliations:** 1 Department for Epidemiology and Preventive Medicine, Professorship for Medical Sociology, University of Regensburg, Regensburg, Germany; 2 Department of Haematology and Internal Oncology, University Hospital Regensburg, Regensburg, Germany; 3 School of Medicine and Public Health, University of Newcastle, Newcastle, Australia; 4 Hunter Medical Research Institute, Newcastle, Australia; 5 Clinical Research, Design and Statistics, Hunter Medical Research Institute, Newcastle, Australia

**Keywords:** Haematological cancer, Survivorship, Unmet needs, Content analysis, Patient-centred care

## Abstract

**Background:**

Some sub-types of haematological cancers are acute and require intensive treatment soon after diagnosis. Other sub-types are chronic, relapse over many years and require life-long cycles of monitoring interspersed with bouts of treatment. This often results in significant uncertainty about the future, high levels of depression and anxiety, and reduced quality of life. Little is known about how to improve care for haematological cancer survivors. This study explored qualitatively, in a sample of haematological cancer survivors, (i) their unmet needs experienced as a result of their disease and treatment; and (ii) strategies that may help address these needs.

**Methods:**

Semi-structured interviews were conducted with 17 adult haematological cancer survivors. Data was analysed using qualitative content analysis. The Supportive Care Framework guided data collection and analysis.

**Results:**

Participants had a mean age of 57 years (SD 13). Most were male (*n* = 10, 59%). Five themes emerged from the data: (i) changes in unmet needs across the care trajectory (with greatest unmet needs experienced soon after diagnosis, at discharge from hospital and with cancer recurrence); (ii) informational unmet needs requiring improved patient-centred communication; (iii) uncertainty about treatment and the future; (iv) coordinated, tailored and documented post-treatment care planning as a strategy for optimal care delivery; and (v) ongoing support services to meet psychosocial and practical unmet needs by involving peer support, less bureaucratic transport services and flexible work arrangements.

**Conclusions:**

To our knowledge, this is the first qualitative investigation using the Supportive Care Framework to explore unmet needs of haematological cancer survivors. Our findings offer fresh insights into this important area of study. Written, take-home care plans which provide simple but tailored guidance on where to seek additional support may help decrease uncertainty and feelings of vulnerability post-treatment for adult haematological cancer survivors. Future research should further develop and test strategies aimed at addressing unmet needs of haematological cancer survivors identified in this study.

**Supplementary Information:**

The online version contains supplementary material available at 10.1186/s12913-020-05927-7.

## Background

### Patient-centred cancer care is considered optimal care

Patient centred care is health care that is based on the needs, preferences and values of each individual patient [1]. It has been consistently recognised as a core component of high-quality cancer treatment and support services [2, 3]. Patient-centred care is particularly important in cancer care as an increasing number of cancer treatment decisions are probabilistic which can create ambiguity and uncertainty among doctors and patients [4]. Also, many cancer patients are anxious and distressed when facing the threat of their disease and the healthcare options available to them [5]. Optimal patient-centred cancer care ensures that cancer patients receive evidence-based and holistic care that is tailored to each patient’s needs and circumstances [2]. This can help maximise patient outcomes [6].

### The supportive care framework to help understand patients’ unmet needs

To provide optimal, patient-centred care to all cancer survivors, we must first identify what survivors would like help with. Unmet needs refer to situations where there is a mismatch between the support a patient requires to meet their concerns, and the services the patient receives [7]. One framework particularly suited to examining patient unmet needs is the *Supportive Care Framework* that conceptualises the help and services cancer patients may require [8]. The *Supportive Care Framework* theorises that a diagnosis of cancer may affect a person’s ability to meet and satisfy their own needs across multiple domains of life, potentially resulting in increased feelings of distress and decreased psychosocial wellbeing [8]. A patient may experience a variety of changes that impact on their needs and require additional support outside their own resources and beyond those offered by routine cancer care [9]. The *Supportive Care Framework* conceptualises seven domains that impact on patients’ and their support persons’ needs, i.e. physical, emotional, social, psychological, spiritual, informational and practical changes [10]. According to the framework*,* it is estimated that all cancer patients will require minimal supportive care in the form of routine assessment, provision of information and good communication [8, 10]. However, up to 50% may require specialised or even intensive support [8, 10].

### Haematological cancer survivors have unique unmet needs

Haematological cancers have wide-reaching impacts on physical, emotional and social wellbeing [11, 12]. However, several features of haematological cancers’ etiology and treatment result in unique needs of haematological cancer survivors compared to their solid tumour cancer counterparts [11, 13]. Common treatment options, such as bone marrow transplantation, peripheral blood cell transplantation and high dose chemotherapy, are lengthy, invasive, and often lead to debilitating side-effects, including extensive comorbidities and serious adverse drug reactions [14–16]. Some sub-types are acute, fast developing, often with poor prognosis that require intensive treatment soon after diagnosis [17, 18]. Other types are chronic and relapse over many years, resulting in significant uncertainty about the future, higher levels of depression and anxiety, and reduced quality of life [19–21]. Many of these chronic haematological cancers require life-long cycles of monitoring interspersed with bouts of treatment [11, 22].

Thus, the clinical courses of haematological cancers are highly variable requiring tailored care that is not always being delivered in clinical practice [23]. For many survivors, their disease becomes a life-altering condition with lengthy treatments and a number of factors impeding on their quality of life, such as excessive fear of recurrence, unemployment and social isolation [24]. Many survivors of haematological cancers require frequent and long-term follow-up care and have to manage their symptoms and side-effects at home, often with insufficient outside support [12]. People affected by haematological cancers also experience higher levels of psychological morbidity often reporting symptoms of anxiety and depression, compared with patients diagnosed with solid tumours [25]. Despite this, relatively few studies have investigated the unmet needs of haematological cancer survivors [26, 27].

### We need in-depth understanding of haematological cancer survivors’ unmet needs

Recent, large-scale quantitative studies have identified the greatest concerns of haematological cancer survivors, using rigorous, standardised measures of unmet needs [26, 28]. The most prevalent unmet needs of haematological cancer survivors relate to: physical symptoms such as feeling tired and coping with having a bad memory or lack of focus; psychological concerns such as feeling worried, anxious or stressed; social concerns such as dealing with the expectations of others, or finding others in a similar situation to talk to; and practical concerns such as finding accessible car parking at the hospital clinic or treatment centre [23, 24, 29, 30]. Furthermore, associations have been found between certain sociodemographic and disease characteristics, such as cancer sub-type, age, financial burden and rurality, and the most prevalent needs experienced by haematological cancer survivors [22, 31]. For example, survivors who had relocated due to their cancer, had used up their savings and who had above normal symptoms of depression and stress had statistically significantly higher odds of reporting more unmet needs [22]. The existing quantitative data only provides limited detail about the meanings, contexts and interrelations of unmet needs from a patient perspective, which is a recognised component of medical care and the doctor-patient relationship [32, 33]. Also, although a number of needs have been identified, little is known about how to address them in routine care [34, 35]. If we are to deliver patient-centred care, we need a more in-depth understanding of the unmet needs haematological cancer survivors experience, the factors that may influence these needs, and the type of support survivors believe may best address these needs. Qualitative research is particularly suited to help fill this gap as it uses open-ended discussion to explore in-depth people’s experiences and perceptions [36].

Yet few qualitative studies have looked at haematological cancer survivors’ unmet needs [35], with much of this research focused only on specific areas of unmet needs, such as spiritual needs [36–38]. For example, McGrath has used qualitative methods to investigate the notion of spirituality among survivors of haematological cancers and how experiencing a serious illness can create potentially positive outcomes for this group [37, 38]. Other qualitative studies explored post-treatment experiences and support needs of survivors of haematological cancers [35, 39, 40]. However, the findings of these studies are limited to survivors of lymphoma or leukemia. A number of studies examined the meaning of the term “survivor” and the views of healthcare providers on optimal survivorship care but did not focus on survivors’ perspectives [41, 42]. Laidsaar-Powell et al. recently conducted a review of qualitative research on adult cancer survivors and found that compared to other common cancers with relatively high survival rates, such as breast or prostate cancer, little is known about the perceptions and experiences of survivors of haematological cancers [43].

## Aims

This Australian study aims to extend and complement previous studies by exploring in-depth:

(i) the unmet needs experienced by haematological cancer survivors recruited from one Australian state as a result of their disease and treatment.
(ii) patient-identified strategies that may help address these unmet needs.

## Methods

### Study design

A qualitative sub-study of a national, cross-sectional survey was conducted in Australia between September 2011 and April 2012 [29]. Participants were a purposeful subsample of adult haematological cancer survivors recruited from one Australian state cancer registry, who had previously participated in a quantitative survey assessing their unmet needs and psychological wellbeing [31, 44].

### Setting and participants

Participants of this study were a subsample of adult haematological cancer survivors taking part in a larger national, quantitative survey study assessing the unmet needs and psychological wellbeing of Australian haematological cancer survivors [45]. For this specific sub-study, participants were recruited from one Australian state cancer registry [45]. Survivors were eligible for the larger study if they were aged 18–80 years at time of recruitment and diagnosed with an ICD-10 or ICD-0-3 defined haematological cancer in the past three years prior to initial recruitment. As permitted by legislation and Human Research Ethics Committee approval participants were contacted directly by the cancer registry without consent. An invitation letter along with a study package was mailed to survivors, including an information statement, survivor questionnaire, nonparticipation form, a brochure explaining the cancer registry, reply-paid envelope, and a questionnaire package for their principal support person. A reminder letter and an additional study package was sent to non-responders approximately four weeks later. Ethics approval for this larger study was obtained from the University of Newcastle Human Research Ethics Committee and the relevant ethics committees associated with each cancer registry. Of the 732 eligible survivors, 268 returned a completed survey resulting in a consent rate of 37%. 715 participants completed the survey and were included in the larger study. The flowchart indicating the recruitment of the 715 haematological cancer survivors is published elsewhere [22].

Survivors were invited to take part in this sub-study if they had returned a completed survey for the cross-sectional study, had consented to the researchers contacting them about future research and were still alive. Eligible survivors were sent a study information pack by mail, which included an information letter and consent form. Survivors were asked to indicate whether they agreed to be involved in the qualitative sub-study. Survivors returned a written consent form via a reply-paid envelope. Consent was obtained via return of a written consent form where they indicated their contact details and preferred day and time of contact. In the information sheet participants were informed that if they do not complete and return the consent form within two weeks they would be sent a reminder. They were asked to return a blank consent form should they do not wish to be sent a reminder. Survivors were also told that if a blank consent form was returned no further contact was made with them regarding this study.

Purposeful sampling was used by the research team to identify and recruit survivors who had reported a high level of unmet needs on the cross-sectional survey. This sampling strategy aimed to capture the meanings, contexts and interrelations of the greatest unmet needs experienced by survivors and to study the variety of factors and circumstances which might influence these needs. The authors acknowledge that haematological cancers are a diverse group of cancers inclusive of over 90 sub-types with considerable variability in symptoms, aggressiveness and treatment options. While a number of studies have focused on specific sub-types, we intended to be inclusive rather than restrictive by utilising the USA *National Cancer Institute’*s definition of a cancer survivor as someone “from the time of diagnosis through the balance of his or her life” [46]. By purposefully sampling those survivors who reported high levels of unmet needs on the quantitative survey, the current study provides an opportunity to explore the issues which are most pressing and which can guide clinical practice and resource allocation.

### Ethical approval

This study was approved by the University of Newcastle Human Research Ethics Committee (approval number H-2009-0032) and the relevant ethics committee associated with the cancer registry.

### Data collection

Interviews were conducted by one researcher (either AH2 or FT) using a semi-structured interview guide which was developed for this study (see Additional file 1). The interview guide involved open-ended questions to elicit narratives from participants. It was designed to assess the following main concepts (i) the nature of unmet needs experienced by survivors as a result of their haematological cancer (i.e. asking participants about what they needed help with, when they needed help and whether they received the help they required); (ii) reasons for these needs (e.g. lack of support services, coordinated care or involvement in decision making); (iii) difficulties with addressing these unmet needs; and (iv) how care could be improved. The interview guide was informed by the Supportive Care Framework, principles of patient-centred care and the results of the cross-sectional survey [29]. A research team involving experts in the areas of psychology and health behaviour science developed the question guide through an iterative process with review and feedback from members of the research team. The guide was piloted with a haematological cancer survivor and their support person. It was updated based on their feedback to address any concerns they may have had. The interview guide was designed to be flexible with questions and prompts used to help elicit topic areas not initially spoken about by the participant. Interviews were recorded and data collection was stopped when data saturation was perceived to be reached [47].

### Data analysis

Qualitative content analysis was considered most appropriate as it allows to systematically describe and interpret the data and to provide a structured approach to study the contexts, meanings and interrelations of survivors’ unmet needs [48]. This helped build a conceptual map and theoretical understanding of unmet needs and areas of support, and how to help address these [49, 50]. All interviews were transcribed verbatim. Codes were then assigned to the data by reading each transcript line by line, and examining, comparing and categorizing its content in order to apply a paraphrase or label (a “code”) that described what was interpreted in the passage as important.

Initially, an inductive qualitative content analysis approach was followed to minimise bias and ensure all relevant codes were captured. This was achieved by employing techniques such as: a) summarising the data to reduce the material in such a way that the essential contents remain; b) explication of data to provide additional material on individual doubtful text components to increase understanding and interpreting of particular passages of text; and c) structuring the data to filter out particular aspects of the material [50]. The unit of analysis was each individual interview. Thus, interviews were first coded separately and then compared with each other [36].

Transparency was achieved by maintaining a journal of reasoning and additional ideas regarding the coding process. This strategy has been successfully used in numerous other studies to facilitate the reconstruction of the analysis and provide justification for the analytical steps undertaken [51, 52]. The journal contained investigations of a code, theme or problem, involving thoughts on emerging issues in the data. This helped better capture the analytic process and discover, develop, and formulate codes, categories and themes. Codes were frequently compared with each other and parts of the material were recoded if necessary. This was used as an intra-coder agreement test and additional measure for reliability [53].

Codes that shared a commonality were then grouped into categories to develop more abstract categories and an initial coding frame [54]. Codes and categories were grouped around the domains of Supportive Care Framework. If a category did not fit into any of the framework’s domains, a separate domain was developed to ensure all data was captured which allowed us to validate and extend conceptually the underlying theoretical framework. Based on the categories, we generated threads of meaning across categories (i.e. “themes”). Data analysis thus aimed to capture both manifest and latent content of the interviews [36]. Manifest content refers to objective evidence that can be directly seen in the data, i.e. visible, obvious components, such as specific words. Latent content refers to more subjective evidence which requires interpretation of the underlying meaning of this content [54]. Initial coding was conducted by one researcher (AH1) and double-checked by another (EM). The categories and coding frame derived from the data were discussed between all members of the research team. This was judged as the most appropriate and feasible inter-coder agreement test to give a measure of objectivity. Patient characteristics are presented using appropriate summary statistics. Rurality was defined based on the Accessibility and Remoteness Index of Australia (ARIA+) [55], with survivors with postcodes classified as outer regional, remote and very remote defined as “rural” and postcodes classified as inner regional and major cities defined as “urban” [56].

## Results

### Sample characteristics

Forty-one patients were approached, the consent rate was 41%. The mean interview duration was 26 min. As shown in Table 1, the majority of survivors were male (*n* = 10, 59%) and diagnosed with Non-Hodgkin’s lymphoma (NHL). Patients had a mean age of 57 years (SD 13).

**Table 1:** Participants’ sociodemographic and disease-related characteristics

Characteristic	Participant (*n* = 17)
Age in years, mean (SD)	57 (13)
Range	19–76
Gender	
Male	59% (10)
Female	41% (7)
Diagnosis	
Non-Hodgkin’s lymphoma (NHL)	59% (10)
Other lymphoma	12% (2)
Leukemia	18% (3)
Myeloma	12% (2)
Time since diagnosis	
0–12 months	30% (5)
1–2 years	47% (8)
More than 2 years	24% (4)
Rurality	
Urban	82% (14)
Rural	18% (3)

### Main themes

Five themes emerged and these are described below: i) changes in unmet needs across the cancer care trajectory; ii) lack of information and involvement in decisions requiring patient-centred communication; iii) uncertainty about treatment and future as areas of concern; iv) coordinated and documented post-treatment care planning; and v) ongoing support services to help meet psychosocial and practical needs. Data saturation was perceived to be reached after having analysed 14 interviews. The following interviews did not provide new categories but helped revise and confirm the existing coding scheme. Table 2 shows how these themes correspond to the domains of the Supportive Care Framework.

**Table 2:** Themes derived from the data, domains of the Supportive Care Framework and suggestions for how the identified needs could be met

Theme	Domains of the Supportive Care Framework	Suggested interventions for routine care
Changes in unmet needs across the cancer care trajectory	Psychological, emotional, informational and practical	Improved patient-centred communication and psychosocial support during the time of diagnosis, cancer recurrence and discharge (e.g. with the help of interactional skills training for clinicians or interactive eHealth or mHealth applications)
Lack of information and involvement in decisions requiring patient-centred communication	Informational, psychological and emotional	Improved patient-centred communication (e.g. by providing more tailored medical information and help with involvement in decision making)
Uncertainty about treatment and future as areas of concern	Informational, psychological, emotional, social and spiritual	Improved patient-centred communication and psychosocial support (e.g. through access to peer support)
Coordinated and documented post-treatment care planning	Informational, psychological, emotional, physical and practical	Provision of care coordinators and written, take-home care plans, tailored to survivors’ individual circumstances and providing guidance on the prescribed steps of care
Ongoing support services to help meet psychosocial and practical needs	Informational, psychological, emotional, social and practical	Provision of: i) further information and referral to less bureaucratic transport services for survivors and support persons; ii) face-to-face peer support sessions occurring on a regular basis and in different locations to maximise survivors’ and their support persons’ ability to attend; and iii) assistance with making flexible work arrangements

### Changes in unmet needs across the cancer care trajectory

Participants reported gradual changes in the nature and intensity of their perceived levels of need for help along the care pathway. Many survivors indicated that the climax of their level of need occurred just after receiving their diagnosis, given the shock of being diagnosed with a life-threatening illness, often despite a perceived lack of symptoms. Participants felt that at this point, their healthcare team could have better informed them about the nature of their condition and next steps of planned care. Many survivors reported a lack of medical information, for example in terms of treatment goals and side-effects, and a lack of psychological support to help them put their diagnosis in context and understand *“what’s going to happen and why it’s happening”* (patient 1).

*I guess at the very beginning is when you probably need it [=support by the healthcare team] most because that’s when you probably as I said in my case you’re almost in a state of shock. You talk to doctors and they’re all a bit matter of fact and clinical about things […]* *So having a more human touch would be nice. You do feel you’re a bit on your own or it’s between you and your family and so forth. That early stage when you’re coping with the reality of a diagnosis I think it’s probably when you need the most support honestly. (patient 2).*

A number of survivors reported another escalation in perceived need for help when their cancer recurred. They often felt increased anxiety and reported tendencies to pessimism and catastrophizing after experiencing a recurrence. Survivors further indicated a need for help with accessing and navigating healthcare services post discharge from hospital. Many reported feeling left alone after leaving the hospital, having little time with their treating clinician to discuss their care and having difficulties with remembering and adhering to their prescribed care. For example, they struggled with keeping track of their medications or accessing help from allied health professionals to manage their symptoms.

*There was no after care. As soon as treatment finished and I was pretty much off all meds and my final check-ups were done, it was kind of like I was just thrown back into the real world after being in a little bubble in hospital for so long. (patient 3).*

*Not just sitting there for a month wondering what on earth was going on. That was my major concern and has been all the time because you only get 15 min with the oncologist and he can’t answer all your questions. You don’t even think of what questions to ask obviously. (patient 4).*

### Lack of information and involvement in decisions requiring patient-centred communication

A number of survivors indicated not receiving sufficient or insufficiently tailored information to help them understand their condition and care. Comprehensive, patient-centred communication was seen to be a key component of optimal care. Conveying empathy and delivering tailored information in lay language were considered particularly important to help survivors and their support persons cope with their disease and treatment.

*[W] hen the specialist sat down for an hour and just said righto, we’ll go through it and I’ll explain everything in layman’s terms, not doctors terms but just normal every day terms. I think that was one of the best parts of it. (patient 5).*

Survivors appreciated being given comprehensive verbal information by their treating physician supplemented by written take-home information which would allow them to digest and enhance the information they received and consider questions they may later have. Survivors wished for respectful communication, for example, by having their physician talk to them in a room that ensured visual and auditory privacy. Many survivors wanted their clinician to adjust the breadth and depth of the information they provided according to their needs and wishes. There was a continuum of survivors’ perceived level of involvement in decision making which was influenced by a number of factors. Despite this, being involved in decision making was commonly seen as a coping mechanism which gave survivors a sense of control over their situation, increased their reassurance about the “right” treatment approach and helped them develop a positive attitude towards care.

*I guess we were reasonably informed although I’m not sure that we were given a whole lot of choice. The doctor would recommend that this is the way forward. He doesn’t say whether you’ve got the choice of A, B and C really so much, but based on our own research I think we understood the process a little bit. (patient 6).*

*Because I think that makes you more positive and comfortable in what you’re doing anyway if you feel you’re involved in it. (patient 7).*

Given the rareness of their condition, some survivors appreciated receiving guidance by the treating physician on where and how to seek additional information, and how to understand and interpret this information. This helped them distinguish between anecdotal and scientific evidence and focus on accurate, up-to-date information that was applicable to their individual circumstances. Many felt that this allowed them to more actively participate in discussions regarding their care.

*There is a lot out there and I guess it [=receiving trustworthy information] allows you to know what questions to ask or think about. You’re never quite sure when you read all this information how much of it is fact and how much of it might be personal to some other person. (patient 2).*

*[T]here’s a wealth of information on the internet but not all of it is current and not all of it is relevant and some of it’s not necessarily accurate. (patient 8).*

### Uncertainty about treatment and future as areas of concern

The rareness of their disease and the perceived “experimental nature” of some treatments also led to many survivors’ perception that there is a lack of evidence available for clinicians to choose the best treatment approach. Some survivors also struggled with the limited treatment options available to them. They perceived blood cancers to be less “tangible” than solid tumours as they cannot be treated with options relevant to most other cancers, such as surgery or radiotherapy. Many participants highlighted that each patient may respond to treatment differently and that their uncertainty about what to expect from their treatment increased their anxiety and distress related to their condition. Survivors felt that it was hard for them to meet peers who have the same (or a very similar) condition and may be willing to share their knowledge of and experiences with care. Given the lack of peer support, survivors often felt lonely and uncertain about the likely short- and long-term impacts of their disease and treatment.

*The doctors themselves are almost working on trial and error. They don’t quite know what is the correct treatment for everybody and it seems to be try a bit of this and see how it goes. You feel like you’re part of an experiment sometimes. […] I guess the issue with this illness is that it’s fairly unknown because most people have never even heard of it […] It’s the difference in my mind between the solid tumours and the blood cancers is that for the tumour you’ve got possibly an option of surgical intervention where you can actually go in, and get the thing and you radiate it or cut it out […]*. *(patient 9).*

*I suppose the biggest thing is you feel alone with it. (patient 1).*

Many survivors contemplated the direction of their future lives. For example, some survivors were unsure about whether to continue their professional career or take-up further education. These, *“philosophical challenges”* (patient 2) often continued for years post-treatment and acted as an impediment to re-entering “normal” life. Many survivors’ feelings of uncertainty continued throughout the disease trajectory.

*I never did further study because when you’re having treatment and everything, when you finish up, the doctors go alright, you need to watch out for this, this and this and this. Then you’ve got five years before you should really think about doing anything because in that five years you’ve got this per cent chance that it could come back or something else could come back. (patient 3).*

### Coordinated and documented post-treatment care planning

Many survivors required long-term follow-up care to manage their symptoms and medications. Participants expressed a need for anticipatory, coordinated, multidisciplinary supportive care post discharge. However only some survivors were given a written, take-home care plan, tailored to their individual circumstances which provided guidance on the steps of care they were prescribed. Such a care plan was seen as a facilitator for effective self-management of care, for example by helping survivors remember when and how to take their medications and allowing them to better anticipate their next appointments and schedule these appointments in a way that allows them to keep other commitments (such as work-related or family commitments).

*[A] structured treatment plan is very important and I think that is actually a help because you can then go to your employer and say look here’s my treatment plan. I’m expecting to be off work or can I do part time for this period of time. Those sorts of questions are necessary. (patient 3).*

A number of survivors said that a tailored care plan could provide them with a feeling of certainty about what is going to happen, even if the care plan may need to be changed due to changes in their health status. A care plan was also perceived as a guide for healthcare providers to talk to survivors about potential symptoms, such as fatigue or nausea, which feelings are considered “normal” and when to seek additional help. It could further be used to explain changes in treatment regimens and inform about potential side-effects of treatment, seek practical advice on symptom management and become more involved in treatment decisions. Some survivors who did not receive a written care plan reported making up their own instructions or diaries to keep track of medications and hospital visits.

*I just have to judge when we were going to be there [=at the hospital] next to get the anti-rejection drugs. So that’s the only hassle. […] I made up a form. I put it on the computer. I used to keep a stock level. Every week I’d go and check what I had, when my next visit was going to be. If it’s going to be, say, 42 days, so I needed at least 42 tablets for this […] and it also made it easier for him [=the treating physician] because he didn’t have to write it down every time I visited. (patient 10).*

*My wife writes everything down in a diary. It’s okay for her, she writes everything down, the times [=of appointments] and that, because I do forget myself. (patient 11).*

Survivors indicated that a tailored care plan could be delivered during or after the consultation with their physician. Some also suggested to implement a standardised programme with follow-up face-to-face meetings or calls provided by care coordinators who use a standardised checklist to inform about all potentially relevant support services and how to access them. Participants further suggested that their care plan could include a compendium of contact details of these support services to help ensure survivors know when and where to seek additional support, such as transport or cleaning services. Such support was seen to help bridge the interim time between consultations with their physician. Survivors also emphasised that this may help them remember and adhere to their care and assist in overcoming their feeling of being overwhelmed.

*[T] hey say you can ring the cancer helpline but you’re too stunned. You have no idea what’s going to happen. I think it would have been a good idea if perhaps, once I was diagnosed, there was a register where my doctor automatically puts you on straightaway, and they can ring you up within the next day or two and say, look we’re here, can we help you through this? […] I see him [=the treating physician] in a fortnight, and it’s that interim time where I think a little bit more information would have been helpful. (patient 12).*

*[S] o that every patient who’s going through chemotherapy, on the first week of contact, have someone ring them up and say do you have someone to clean your house, do you have someone to give you food, do you have emotional support, to do a checklist to make sure that if you get people like me who have no one, then you can act. (patient 14)*

### Ongoing support services to help meet psychosocial and practical needs

A number of survivors recommended that non-profit organisations could provide more information and peer support sessions to allow survivors to receive further advice on their care, share experiences with others, overcome loneliness, and provide a sense of community. Survivors said that it would be important for these sessions to occur face-to-face, on a regular basis and in different locations to maximise survivors’ and their support persons’ ability to attend.

*I joined a self-help little group that we used to go and introduce ourselves and talk about our attitudes and what’s happening. It was quite good just talking around the table. Anyone who came in new, this positive attitude rubbed off on them. […] [T] here were so many different sort of treatments that people could talk about it and talk about their experiences and then I thought, oh well, I wasn’t such a dumb-dumb after all. Different things happen to different people in different ways. That’s good for someone who doesn’t understand anything medical-wise. (patient 15).*

*[P] otentially multiple locations rather than just one. It’s all very well to have one if you’re only getting half a dozen people but if you can get half a dozen people at two locations well that’s better still. (patient 2).*

Peer support sessions were seen particularly important as some survivors reported being reluctant to speak to their support persons about their needs and wishes, given that they did not wish to cause them any additional burden. They appreciated if their support persons were given the opportunity to receive respite and peer support, in order to meet their own psychosocial needs.

*Yeah. I feel really alone about it because I don’t talk about it to the family type of thing. I just clam up about it. I won’t talk to them because they get upset. (patient 1).*

A number of participants reported having to travel long distances to their treatment centre, resulting in considerable travel times and costs, and time away from their support persons. Some survivors reported having support persons who were still working full-time, thus making it harder for them to bring the person they care for to the clinic. Many indicated that services, such as assistance with transport and overnight accommodation for support persons, would help them access care, but there was also a lack of knowledge among many survivors on which services are available and how to use them.

*Well, we live in the country and we live a long way from any sort of local help. It’s our fault, nobody else’s, but it was awkward. I mean, I have to go 120 km just to get blood tests. […] My husband had to have an awful lot of time off work to take me in [=to the hospital], get blood tests and then come with me for chemo […]. (patient 12).*

Existing transport services were criticized by many survivors for being costly, only running to certain destinations and for having to be shared with others. Some survivors pointed out bureaucratic barriers to accessing transport support services. For example, they reported not getting reimbursed until the end of treatment and having difficulties with gathering all required paperwork for their reimbursement. This was perceived to be particularly difficult given the array of things they felt they had to attend to ensure they adhered to their prescribed care. Further, transport services often did not include services for support persons who may not be able to travel to the treatment centre themselves.

*One of the biggest stresses that we’ve had has been with PATS, Patient Assisted Travel Scheme. […] Indeed rather than trying to argue with them that, oh look, I need a flight home on such a such date and then I’m going to have to come back down again. Yeah, it’s easier just to do it yourself and ignore it. […] Well my wife for example keeps saying she wishes that the people who were in the PATS office had been through this sort of circumstances themselves. That then they might have a little more compassion. It’s a bit harsh but I know exactly where she’s coming from. (patient 2).*

A number of survivors reported that being able to continue working gave them a sense of purpose, provided distraction and financial security, especially given that some treatment required considerable out-of-pocket costs. Some survivors reported loneliness and boredom when having to stay at home, particularly when their support persons were at work. Receiving help with making flexible work arrangements for themselves and their support persons was thus seen to help reduce the impact of the disease on their lives and meet their psychological and practical needs.

*I think you would go crackers, especially when you’re home most of the day and when everyone goes to … I mean, all the kids are grown up now, so there’s only me and my wife here. So when she goes to work it can get a little bit boring. (patient 11).*

*I’ve got a really understanding boss that if he sees I’m looking really crook with it, he’ll just say go home, which is really good. Without that I’d be buggered. (patient 1).*

*I think my recommendation for other people in a similar situation would be look, talk to your employer. See if you can do part-time work. Something to keep yourself interested. To give yourself an interest outside yourself. (patient 2).*

## Discussion

People affected by haematological cancers still experience higher levels of psychological morbidity often reporting symptoms of anxiety and depression, compared with patients diagnosed with other common cancer types [25]. However, compared to other common cancers with relatively high survival rates such as breast or prostate cancer, little is known about the needs of survivors of haematological cancers and how to meet them [43]. To our knowledge, this is the first qualitative study to employ the Supportive Care Framework to provide an in-depth understanding of haematological cancer survivors’ needs and how to overcome them. Table 2 summarizes the themes, domains of the Supportive Care Framework to which the themes correspond, and suggestions for how the identified needs could be met. The findings of this study suggest five predominant and overlapping areas of concern with some thematic content consistent with the findings of previous quantitative [29] and qualitative [35, 57] studies on unmet needs in this population. These themes cover the domains of the Supportive Care Framework, including psychological, social, emotional, spiritual, informational, physical and practical needs. This highlights the multi-faceted impacts of the disease on survivors, and the need to provide support across all domains of the framework to improve patient outcomes.

As there is still a lack of research on haematological cancer survivors’ care experiences [43], our findings add to the literature by providing fresh insights into the needs of this patient population and how to overcome them. For example, it has been reported that survivors of other cancers also struggle with a lack of practical and psychological support [27, 43]. Given the variation in clinical characteristics across the different sub-types of haematological cancers, the rareness of many of these sub-types and many survivors’ perceptions that blood cancers are more difficult to treat than solid tumours, other aspects we describe in this manuscript are more specific to survivors of haematological cancers. This includes the need for patient-centred communication and tailored, coordinated and documented post-treatment care planning. These needs have been reported less frequently for survivors of other cancers [27, 43], indicating a unique need for survivors of haematological cancers.

Referrals to peer support, less bureaucratic transport services and flexible work arrangements were seen as further enablers for optimal care delivery. Taken together these strategies could help provide patient-centred care that is responsive to the specific needs of individual patients Including the provision of emotional support and information and communication that enables patients to understand their options and make informed decisions regarding their care [21, 22]. Optimal patient-centred care thus involves understanding each individual patient’s personal meaning of the illness and building a relationship between doctor and patient that is based on mutual understanding, empathy and trust [23, 24].

Haematological cancer survivors described different unmet needs at different time points along the cancer care trajectory, with immediate post-diagnosis identified by many as a time when unmet needs are most profound. A qualitative study of non-Hodgkin’s lymphoma patients similarly reported that the period around diagnosis was a crucial time psychologically [57]. Adapting to a cancer diagnosis can be a prolonged process with the first year after diagnosis commonly recognised in the wider cancer literature as the most difficult [58].

The need for information related to diagnosis and treatments was viewed by survivors as an important area of concern. Our findings suggest that the way that information is communicated is as important, if not more, than the volume of information regarding diagnosis and treatment. This is consistent with findings from previous research [57, 59]. Simple language, multiple formats (e.g. verbal and written) and empathic delivery are key factors in how well survivors feel supported and in reducing unmet needs [57]. Additionally, information regarding prognosis and needs relating to spiritual concerns and future life directions are important for survivors. Information provision should be tailored to individual survivors’ needs and preferences [60]. The importance of tailoring information may be particularly pertinent to haematological cancer survivors, with a number reporting receiving insufficient specific information about their sub-type of haematological cancer. General practitioners and other healthcare providers are often not very familiar with over 90 different sub-types of haematological cancers [61–63]. Thus, inadequacies in the provision of information persist which highlights the need for clear pathways to direct haematological cancer survivors to resources that provide more specific information about their form of cancer. Asking survivors about the content and amount of information they would like to receive may provide a first step in this direction and help structure communication and ensure optimal information provision [64, 65]. Tailored information provision could also be achieved by referring patients to trustworthy online information about their particular sub-type of cancer or their psychosocial concerns. More innovative information provision strategies, such as improved interactional skills trainings or interactive eHealth or mHealth applications may further facilitate the provision of tailored information [66].

Another critical phase in the cancer care trajectory identified in both the present investigation and in previous research [57] was the end of treatment. Participants here described a perceived withdrawal of ‘centralised’ care and regular contact with clinicians, moving to a fragmented and poorly communicated approach to their ongoing care post-treatment. Integrated and centralised care is a key component of providing optimal patient-centred care. Our findings indicate that some survivors may benefit from a clearly written, take-home care plan that reflects co-ordinated and multi-disciplinary supportive care tailored specifically to their individual needs. Failure to provide survivors with an ongoing care plan is likely to lead to confusion and distress, impacting on both their psycho-social wellbeing and their capacity to effectively self-manage their care at home. Uncertainty, vulnerability and a feeling of neglect among survivors resulting from “gaps” in post-treatment care have been acknowledged in the wider cancer literature [67]. This is particularly pertinent for haematological cancer survivors, especially those with chronic forms of cancer who are consistently going through monitoring followed by active treatment [68].

Finally, survivors appear to have a number of concerns regarding identifying and accessing cancer care support services, both formal and informal, on an ongoing basis. Due to the rareness of their particular type of cancer, inadequate transport and long distances involved, many survivors are unable to engage with support groups to address their need for social support [69, 70]. To diminish feelings of isolation and help make sense of their experience, survivors often find it valuable to form connections with other survivors [57]. However, many survivors are not made aware of, or referred to, appropriate non-profit cancer support services [71].

### Limitations

This study had a number of limitations. Our sample did not involve all types of haematological cancers and may thus not mirror the experiences and preferences of survivors from types of cancers not included in this study. However, our sample included different types of lymphomas and leukemias and the sample size was larger than previous qualitative studies on this population [57]. Second, as most participants were greater than one year since their diagnosis, survivors recall of their cancer experience shortly after diagnosis may be biased by those with particularly high levels of unmet needs. However, it allowed us to identify unmet needs at different stages of the trajectory. Third, participants were recruited from one Australian state and their experiences may be different from survivors in other states. Also, given differences in healthcare systems and services their experiences may be different from those of cancer survivors in other countries. Despite this, our findings highlight some unmet needs that have been reported for similar patient populations of other countries [21]. Thus, our suggestions for how to improve care may be applicable to other settings. Future research should further examine the applicability and usefulness of our findings for other jurisdictions. Finally, it may not be feasible to implement survivors’ exact suggestions to address their needs due to finite resources. However, the data reported in this study can be used to develop strategies that take into account survivor needs combined with pragmatic resource constraints.

## Conclusions

Understanding the critical periods, specific types of unmet needs and strategies to help address these needs can facilitate more effective models of care and support services for haematological cancer survivors. This study has indicated that survivors want comprehensive and easily understood information in multiple formats. They want to be able to ask questions and participate in shared decision making with clinicians. Written, take-home care plans providing simple and tailored instructions and guidance on where to seek additional information could help decrease uncertainty and feelings of vulnerability post-treatment. Identification of, and referrals to, appropriate peer support and community groups should be included in care plans with improved communication and co-ordination of care between clinicians, primary care providers and community care services [58]. Future research should develop and test the effectiveness of the strategies identified in this study as being most likely to address the specific unmet needs experienced by haematological cancer survivors.

## Supplementary Information

**Additional file 1.**

## Data Availability

The datasets generated during and/or analysed during the current study are available from the corresponding author on reasonable request.

## Abbreviations

ARIA+: Accessibility and Remoteness Index of Australia
NHL: Non-Hodgkin’s lymphoma
