# Supplementary material for: Use of the supportive care framework to explore haematological cancer survivors’ unmet needs: a qualitative study
Source: BMC Health Serv Res. 2020 Nov 23;20:1062. doi: 10.1186/s12913-020-05927-7 (PMC7686725; doi:10.1186/s12913-020-05927-7)
Supplement: Supplementary file 1 — Additional file 1. [file 12913_2020_5927_MOESM1_ESM.doc]

SEMI-STRUCTURED DISCUSSION GUIDE FOR TELEPHONE INTERVIEWS – PATIENTS

Thank you so much for talking to me today. I very much appreciate you taking the time.

**FIRSTLY, I’D LIKE TO ASK YOU ABOUT YOUR EXPERIENCES WITH BLOOD CANCER.**

Since being diagnosed what have been the top five things that you needed help with?

*(PROMPT: Needs can relate to a number of areas such as:* ***health concerns*** *i.e. diet and nutrition;* ***emotional*** *i.e. worry about the future;* ***practical concerns*** *i.e. assistance with housework;* ***information concerns*** *i.e. details on how to address any side effects you may experience).*

Did you get the help you needed?

If not: What could have been done to give you the help you needed?

What things have you RECENTLY needed help with as a result of your cancer? Did you get the help you needed?

When was the last time you needed help to meet your needs? What were these needs?

During what stage of your cancer diagnosis did you experience the most needs you needed help with? What were they?

**I’D NOW LIKE TO ASK YOU ABOUT THE CANCER CARE YOU RECEIVED.**

Can you tell me about the cancer care you received, such as the treatment and support you were given?

What do you think were the top 5 things that could have been done to improve your cancer care?

What do you think were the top 5 things that were done well during your cancer care?

How were decisions made about your cancer care?

Were you happy with your involvement in the decision making process?

**I’D NOW LIKE TO ASK YOU WHAT YOU THINK BEST POSSIBLE CANCER CARE SHOULD INVOLVE.**

What do you think best possible cancer care should include?

Did you feel you received the best possible cancer care?

**I’D NOW LIKE TO ASK YOU ABOUT SPECIFIC FEATURES OF CANCER CARE.**

**What should doctors and nurses provide during cancer care?**

(PROMPTS: be respectful, manage physical symptoms, manage emotional needs, have good communication skills, be up-to-date about care)

**What types of information should be provided during cancer care?**

(PROMPTS: diagnosis, life expectancy, side effects, what to do after treatment)

**What things should hospitals do to provide quality treatment during cancer care?**

(PROMPTS: short waiting times, provide correct treatment, provide consistent information, eliminate unnecessary tests or treatments)

**What type of support services should be offered during cancer care?**

(PROMPTS: emotional, spiritual, address changes in relationships, practical help with finances, childcare, transport, housework etc)

What should be offered when making major decisions about cancer care?

(PROMPTS: adequate time to discuss treatment options with doctor, access to information about treatment options through an internet website or printed materials, opportunity to review all options with family members at home before final decision, being offered a second medical opinion)

**What things do you think families of cancer patients should be offered?**

(PROMPTS: involvement in treatment decision making, help to manage the patient’s care at home, counselling to help cope, access to other families in the same situation)

**What things do you think are needed for cancer care to be coordinated?**

(PROMPTS: co-ordination when discharged from hospital to home; co-ordination when moving between different hospitals or other health services; co-ordination when moving from the treatment phase to follow up care)

**What things should cancer patients be able to choose?**

(PROMPTS: which hospital provides their treatment, which doctor provides their treatment, to see the same doctor for each appointment)

**What type of care should cancer patients to be able to access?**

(PROMPTS: quality care that is affordable, quality care close to where they live, quality care regardless of whether they have health insurance)

Thank you for taking the time to speak with me today. The time and information you have provided is greatly appreciated. Just before we end this telephone call I would like to offer you the phone number for two organisations that specialise in providing cancer support and information; as sometimes speaking about your cancer experience can raise new questions or cause distress.

- The first number is for the Leukaemia Foundation, which is an organisation which specialises in blood cancer and provides information and support for those affected by blood cancer. Their freecall number is 1800 620 420.
- The second is the Cancer Council Helpline. This is a free, confidential telephone service that is staffed by specialised cancer nurses. Their phone number is 13 11 20.

If you experience any distress related to what we spoke about today or about your experience with cancer, please do not hesitate to contact either of these services.

Thank you again.
